# Weighted inequalities for generalized polynomials with doubling weights

**DOI:** 10.1186/s13660-017-1369-0

**Published:** 2017-04-27

**Authors:** Haewon Joung

**Affiliations:** Department of Mathematics, Inha University, 100 Inha-ro, Nam-gu, Incheon, 22212 South Korea

**Keywords:** 26D05, 42A05, generalized polynomials, Bernstein inequality, Marcinkiewicz inequality, Schur inequality, Remez inequality, Nikolskii inequality, Doubling weights, large sieve

## Abstract

Many weighted polynomial inequalities, such as the Bernstein, Marcinkiewicz, Schur, Remez, Nikolskii inequalities, with doubling weights were proved by Mastroianni and Totik for the case $1 \leq p < \infty$, and by Tamás Erdélyi for $0< p \leq1$. In this paper we extend such polynomial inequalities to those for generalized trigonometric polynomials. We also prove the large sieve for generalized trigonometric polynomials with doubling weights.

## Introduction

A generalized nonnegative trigonometric polynomial is a function of the type

1.1 $$ f(x)=\vert \alpha \vert \prod_{j=1}^{m} \biggl\vert \sin \biggl( \frac {x-z_{j}}{2} \biggr) \biggr\vert ^{r_{j}} \quad (0\neq\alpha\in\mathbb{C}) $$

with $r_{j}\in\mathbb{R}^{+}$, $z_{j}\in\mathbb{C}$, and the number

$$n\overset{\mathrm{def}}{=}\frac{1}{2} \sum_{j=1}^{m}r_{j} $$

is called the degree of *f*.

We denote by $\mathbb{GT}_{n}$ ($n\in\mathbb{R}^{+}$) the set of all generalized nonnegative trigonometric polynomials of degree at most *n* and we denote by $\mathbb{T}_{n}$ ($n \in\mathbb{N}$) the set of all real trigonometric polynomials of degree at most *n*.

In this paper we work on the real line. If $x \in\mathbb{R}$, then

$$\begin{aligned} \biggl\vert \sin \biggl( \frac{x-z_{j}}{2} \biggr) \biggr\vert = & \biggl( \sin \biggl( \frac{x-z _{j}}{2} \biggr) \sin \biggl( \frac{x-\bar{z}_{j}}{2} \biggr) \biggr) ^{1 /2} \\ =& \frac{1}{\sqrt{2}} \bigl( \cosh(\operatorname{Im}{z_{j}}) -\cos(x- \operatorname{Re}{z_{j}}) \bigr) ^{1/2}, \end{aligned}$$

therefore, $f \in\mathbb{GT}_{n}$ can be written as

$$f = \prod_{j=1}^{m} T_{j}^{r_{j} /2} ,\qquad \sum_{j=1}^{m} r_{j}/2 \leq n, $$

where $T_{j}$ is a nonnegative real trigonometric polynomial of degree 1. Many inequalities for generalized nonnegative polynomials are known; see [1].

Note that if $f \in\mathbb{GT}_{n}$ with each $r_{j} \geq2$ in its representation (1.1), then *f* is differentiable for all $x \in\mathbb{R}$.

In this paper we deal with doubling weights and $A_{\infty}$ weights. An integrable, 2*π*-periodic weight function *W* is called a doubling weight if there is a positive constant *L* such that

1.2 $$ \int_{2J} W \le L \int_{J} W $$

for any interval $J\subset\mathbb{R}$, where 2*J* is the interval with length $2\vert J\vert $ ($\vert J\vert $ denotes the Lebesgue measure of the set *J*) and with midpoint at the midpoint of *J*. The constant *L* in (1.2) will be called the doubling constant. A periodic weight function *W* on $\mathbb{R}$ is an $A_{\infty}$ weight if for every $\epsilon>0$, there is a $\delta>0$ such that

$$\int_{E} W\geq\delta \int_{J} W $$

for any interval $J \subset\mathbb{R}$ and any measurable set $E\subset J$ with $\vert E\vert \geq\epsilon \vert J\vert $. Obviously $A_{\infty}$ weights are doubling weights. Many properties of doubling and $A_{\infty}$ weights are studied; see [2].

Weighted polynomial inequalities, such as Bernstein, Marcinkiewicz, Remez, Schur, Nikolskii inequalities, with doubling and $A_{\infty}$ weights were proved by G. Mastroianni and V. Totik in [2], where $L_{p}$ norm is considered for $1\leq p <\infty$. For $0< p\leq1$, Tamás Erdélyi [3] proved such inequalities for the trigonometric case. Recently, it has been proved that inequalities of this kind hold also for more general weight functions, namely for the product of a doubling and an exponential weight (see [4]) and for a class of nondoubling weights (see [5]).

In this paper we show that many weighted polynomial inequalities hold for generalized nonnegative trigonometric polynomials as well. We also prove the large sieve for generalized trigonometric polynomials with doubling weights.

The rest of this paper is organized as follows. In Section 2, we prove the basic theorems which will be used in the proof of weighted inequalities for generalized trigonometric polynomials. In Section 3, we prove Bernstein, Marcinkiewicz, and Schur inequalities for generalized trigonometric polynomials with doubling weights and in Section 4 we prove Remez and Nikolskii inequalities for generalized trigonometric polynomials with $A_{\infty}$ weights.

## The basic theorems

The following theorem is a basic tool in proving the weighted inequalities for generalized trigonometric polynomials. For ordinary polynomials the theorem is proved by Mastroianni and Totik in [2] for $1 \leq p< \infty$, and by Tamás Erdélyi in [3] for $0< p \leq1$. The proof is a modification of their arguments.

### Theorem 2.1

*Let* $0< p<\infty$. *Let* *W* *be a doubling weight*, *and let*

2.1 $$ W_{n}(x):= n \int_{x- 1/n}^{x+ 1/n} W(t)\,dt. $$

*Then there is a constant* $C>0$ *depending only on* *p* *and on the doubling constant* *L* *such that for every* $f \in\mathbb{GT}_{n}$ ($1\leq n \in\mathbb{R}^{+}$) *with each* $r_{j} \geq2$ *in its representation* (1.1) *we have*

2.2 $$ C^{-1} \int_{-\pi}^{\pi} f^{p} W \leq \int_{-\pi}^{\pi} f^{p} W _{n} \leq C \int_{-\pi}^{\pi} f^{p} W. $$

The function $W_{n}$ in (2.1) is continuous and can be approximated by polynomials as follows. If $1 \leq n \in\mathbb{R} ^{+}$, then^1^

$$\frac{1}{2L}W_{n}(x) \leq W_{[n]}(x) \leq L W_{n}(x), $$

uniformly in $x \in\mathbb{R}$, hence, by Theorem 2.2 in [3], for $0< p<\infty$, for each $n \in\mathbb{R}^{+}$ ($n \geq1$) there is a nonnegative real trigonometric polynomial $P_{n}$ of degree at most $( \frac{\log_{2} L}{p} +4) n$ such that^2^

2.3 $$ \bigl(P_{n}(x)\bigr)^{p} \sim W_{n}(x) $$

and

2.4 $$ \bigl\vert P'_{n}(x)\bigr\vert ^{p} \leq C n^{p} W_{n}(x), $$

uniformly in $x \in\mathbb{R}$.

The following lemma plays a crucial role in proving Theorem 2.1.

### Lemma 2.2

*Let* $0< p<\infty$ *and let* *W* *be a doubling weight*, *and*

$$W_{n}(x):= n \int_{x- 1/n}^{x+ 1/n} W(t)\,dt. $$

*Let*

$$0 \leq\tau_{1} < \tau_{2} < \cdots< \tau_{m} \leq2\pi $$

*and*

$$\delta= \min\bigl\{ \tau_{2} - \tau_{1}, \ldots, \tau_{m} - \tau _{m-1}, 2 \pi- (\tau_{m} - \tau_{1})\bigr\} >0 . $$

*Then there is a constant* $C>0$ *depending only on* *p* *and on the weight* *W* *such that for every* $f \in\mathbb{GT}_{n}$ ($n \in\mathbb{R} ^{+}$) *we have*

$$\sum_{j=1}^{m} f^{p}( \tau_{j})W_{n}(\tau_{j}) \leq C \biggl( \frac{bn+1}{2 \pi} + \delta^{-1} \biggr) \int_{0}^{2\pi} f^{p}(x)W_{n}(x)\,dx, $$

*where* $b:= ( \frac{\log_{2} L}{p} +5) $.

### Proof

Applying Theorem 2.2 in [6] to $f^{p} \in\mathbb{GT}_{np}$ with $r=p$ and $\Psi(x)=x$, we have

2.5 $$ \sum_{j=1}^{m} f^{p}(\tau_{j}) \leq \biggl( \frac{n+1}{2\pi} + \delta ^{-1} \biggr) \frac{e(p+1)}{2} \int_{0}^{2\pi} f^{p}(x)\,dx. $$

The polynomial $P_{n}$ in (2.3) has degree at most $( \frac{\log_{2} L}{p} +4) n$, hence, $fP_{n} \in \mathbb{GT}_{bn}$ where $b= ( \frac{\log_{2} L}{p} +5) $. Applying (2.3) and (2.5) we have

$$\begin{aligned} \sum_{j=1}^{m} f^{p}(\tau_{j})W_{n}(\tau_{j}) \leq& C_{1} \sum_{j=1} ^{m} \bigl( f(\tau_{j})P_{n}(\tau_{j}) \bigr) ^{p} \\ \leq& C_{2} \biggl( \frac{b n+1}{2\pi} + \delta^{-1} \biggr) \int _{0}^{2\pi} \bigl( f(x)P_{n}(x) \bigr) ^{p}\,dx \\ \leq& C_{3} \biggl( \frac{b n+1}{2\pi} + \delta^{-1} \biggr) \int _{0}^{2\pi} f^{p}(x)W_{n}(x)\,dx, \end{aligned}$$

which completes the proof. □

As an application of Theorem 2.1 we have the following weighted analog of a large sieve.

### Theorem 2.3

*Let* $0< p<\infty$ *and let* *W* *be a doubling weight*. *With the same notations as in Lemma* 2.2, *there is a constant* $B>0$ *depending only on* *p* *and on the weight* *W* *such that for every* $f \in\mathbb{GT}_{n}$ ($1\leq n \in\mathbb{R}^{+}$) *with each* $r_{j} \geq2$ *in its representation* (1.1) *we have*

$$\sum_{j=1}^{m} f^{p}( \tau_{j})W_{n}(\tau_{j}) \leq B \biggl( \frac{bn+1}{2 \pi} + \delta^{-1} \biggr) \int_{0}^{2\pi} f^{p}(x)W(x)\,dx, $$

*where* $b:= ( \frac{\log_{2} L}{p} +5) $.

### Proof

Applying Lemma 2.2 and Theorem 2.1, we have

$$\begin{aligned} \sum_{j=1}^{m} f^{p}( \tau_{j})W_{n}(\tau_{j}) \leq& C \biggl( \frac{bn+1}{2 \pi} + \delta^{-1} \biggr) \int_{0}^{2\pi} f^{p}(x)W_{n}(x)\,dx \\ \leq& B \biggl( \frac{b n+1}{2\pi} + \delta^{-1} \biggr) \int_{0} ^{2\pi} f^{p}(x)W(x)\,dx, \end{aligned}$$

which completes the proof. □

We now prove Theorem 2.1.

### Proof of Theorem 2.1

We closely follow the proof of Theorem 2.1 in [3]. Let $0< p<\infty$. First we show that there is a constant $C>0$ depending only on *p* and on the doubling constant *L* such that for every $f \in\mathbb{GT}_{n}$ ($1 \leq n \in \mathbb{R}^{+}$) with each $r_{j} \geq2$ in its representation (1.1) we have

2.6 $$ \int_{-\pi}^{\pi} \bigl\vert f'\bigr\vert ^{p} W_{n} \leq C n^{p} \int_{-\pi}^{\pi} \vert f\vert ^{p} W_{n} . $$

In fact by (2.3), there is a polynomial $P_{n}$ of degree at most $N = ( \frac{\log_{2} L}{p} +4) n$ such that

$$\int_{-\pi}^{\pi} \bigl\vert f^{\prime}\bigr\vert ^{p} W_{n} \sim \int_{-\pi}^{\pi} \bigl\vert f ^{\prime}\bigr\vert ^{p} \vert P_{n}\vert ^{p} . $$

Using

$$f^{\prime}P_{n}=(fP_{n})^{\prime} - fP_{n}^{\prime} $$

and $(a+b)^{p} \leq2^{p} (a^{p} +b^{p})$ for any $a, b, p>0$, we have

$$\begin{aligned} \int_{-\pi}^{\pi} \bigl\vert f^{\prime}\bigr\vert ^{p} \vert P_{n}\vert ^{p} \leq& 2^{p} \biggl( \int_{-\pi}^{\pi} \bigl\vert (f P_{n})^{\prime} \bigr\vert ^{p} + \int_{-\pi}^{\pi} \bigl\vert f P_{n}^{\prime} \bigr\vert ^{p} \biggr). \end{aligned}$$

For the first term in the right hand side of the above inequality, we use Bernstein’s inequality (Theorem 10 and its Remark in [1]) for generalized trigonometric polynomials of degree at most $(n+N)$, and (2.3), then we have

$$\begin{aligned} \int_{-\pi}^{\pi} \bigl\vert (f P_{n})^{\prime} \bigr\vert ^{p} \leq& C_{1} (n+N)^{p} \int_{-\pi}^{\pi} \vert f P_{n}\vert ^{p} \\ \leq& C_{2} (n+N)^{p} \int_{-\pi}^{\pi} \vert f\vert ^{p} W_{n}. \end{aligned}$$

For the second term, we use (2.4), then we have

$$\int_{-\pi}^{\pi} \bigl\vert f P_{n}^{\prime} \bigr\vert ^{p} \leq C_{3} n^{p} \int_{-\pi}^{\pi} \vert f\vert ^{p} W_{n}. $$

Since

$$n \sim N= \biggl( \frac{\log_{2} L}{p} +4 \biggr) n, $$

we have, for $0< p<\infty$,

$$\int_{-\pi}^{\pi} \bigl\vert f^{\prime}\bigr\vert ^{p} W_{n} \leq C n^{p} \int_{- \pi}^{\pi} \vert f\vert ^{p} W_{n}. $$

Thus the proof of (2.6) is complete.

Note that the case $1 < p < \infty$ of the theorem follows from the case $0< p\leq1$. In fact, if $1 < p < \infty$ then we may apply the theorem for the case $0< p\leq1$ with *f* and *p* replaced by $f^{p}$ and 1, respectively. Since

$$\frac{1}{p}W_{pn}(x) \leq W_{n}(x) \leq \frac{L^{(\log_{2}p)}}{p} W _{pn}(x), $$

uniformly in $x \in\mathbb{R}$, the case $1 < p < \infty$ of the theorem follows.

So from now on we assume that $0< p\leq1$. Now let *K* be a large positive even integer which will be chosen later, and set $n^{*} = [n]$ and

$$J_{i} := \biggl[ \frac{2i\pi}{Kn^{*}}, \frac{2(i+1)\pi }{Kn^{*}} \biggr] , \quad i=0, 1, \ldots, Kn^{*} -1. $$

Let $\alpha_{i} \in J_{i}$ be a point such that $f(\alpha_{i}) = \max_{x \in J_{i}} f(x)$ and let $\beta_{i} \in J_{i}$ be a point such that $W_{n}(\beta_{i})=\max_{x \in J_{i}} W_{n}(x)$. Let

$$M_{n}= \sum f^{p}(\alpha_{i}) W_{n}(\beta_{i}), $$

where the summation is taken for $i=0, 1, \ldots, Kn^{*} -1$, unless stated otherwise. Now let $\xi_{i} \in J_{i}$ be arbitrary. Using $a^{p}-b^{p} \leq(a-b)^{p}$ for $a \geq b \geq0$, $0< p\leq1$, we have

2.7 $$\begin{aligned} M_{n} - \sum f^{p}( \xi_{i}) W_{n}(\beta_{i}) =& \sum \bigl(f^{p}(\alpha _{i})-f^{p}( \xi_{i})\bigr) W_{n}(\beta_{i}) \\ \leq& \sum\bigl(f(\alpha_{i})-f(\xi_{i}) \bigr)^{p} W_{n}(\beta_{i}) \\ \leq& \sum\bigl\vert f^{\prime}(\tau_{i}) ( \alpha_{i} - \xi_{i})\bigr\vert ^{p} W_{n}( \beta_{i}) \\ \leq& \biggl( \frac{2\pi}{Kn^{*}} \biggr) ^{p} \sum\bigl\vert f^{\prime }(\tau _{i})\bigr\vert ^{p} W_{n}(\beta_{i}) \end{aligned}$$

with some $\tau_{i} \in J_{i}$. Since, uniformly for $x, y \in J_{i}$,

$$L^{-1}W_{n}(x) \leq W_{n}(y)\leq LW_{n}(x), $$

we can continue this:

2.8 $$\begin{aligned} \leq& \biggl( \frac{2\pi}{Kn^{*}} \biggr) ^{p} C_{3} \sum\bigl\vert f'(\tau _{i}) \bigr\vert ^{p} W_{n}(\tau_{i}). \end{aligned}$$

Now we write

$$\sum\bigl\vert f'(\tau_{i})\bigr\vert ^{p} W_{n}(\tau_{i})= \sum _{i=2l} \bigl\vert f'(\tau_{i})\bigr\vert ^{p} W_{n}(\tau_{i}) +\sum _{i=2l+1} \bigl\vert f'(\tau_{i})\bigr\vert ^{p} W_{n}(\tau_{i}) $$

and then applying Lemma 2.2, we have

$$\sum_{i=2l} \bigl\vert f'( \tau_{i})\bigr\vert ^{p} W_{n}( \tau_{i}) \leq C_{4} \biggl( \frac{bn+1}{2 \pi}+ \frac{Kn^{*}}{2\pi} \biggr) \int_{0}^{2\pi} \bigl\vert f'\bigr\vert ^{p} W_{n} $$

and

$$\sum_{i=2l+1} \bigl\vert f'( \tau_{i})\bigr\vert ^{p} W_{n}( \tau_{i}) \leq C_{4} \biggl( \frac{bn+1}{2 \pi}+ \frac{Kn^{*}}{2\pi} \biggr) \int_{0}^{2\pi} \bigl\vert f'\bigr\vert ^{p} W_{n} , $$

hence

$$\begin{aligned} \sum\bigl\vert f'(\tau_{i})\bigr\vert ^{p} W_{n}(\tau_{i}) \leq& \frac{C_{4}}{\pi} \bigl(bn+1+Kn ^{*}\bigr) \int_{0}^{2\pi} \bigl\vert f'\bigr\vert ^{p} W_{n} \\ \leq& C_{4} Kn \int_{0}^{2\pi} \bigl\vert f'\bigr\vert ^{p} W_{n} , \end{aligned}$$

where we assume that $K \geq ( \frac{\log_{2}L}{p}+6) $ so that $bn+1 \leq Kn$ (*b* is defined in Lemma 2.2). Thus, by using the above inequality and (2.6), we can continue the inequality (2.8) thus:

2.9 $$\begin{aligned} M_{n} - \sum f^{p}( \xi_{i}) W_{n}(\beta_{i}) \leq& C_{5} \biggl( \frac{2 \pi}{Kn^{*}} \biggr) ^{p} Kn \int_{0}^{2\pi} \bigl\vert f'\bigr\vert ^{p} W_{n} \\ \leq& C_{6} K^{1-p} n \int_{0}^{2\pi} \vert f\vert ^{p} W _{n}. \end{aligned}$$

Since

$$\begin{aligned} \int_{0}^{2\pi} \vert f\vert ^{p} W_{n} =& \sum \int_{J_{i}} f^{p} W _{n} \\ \leq& \sum \vert J_{i}\vert f^{p}( \alpha_{i}) W_{n}(\beta _{i})= \frac{2\pi}{Kn^{*}}M_{n} , \end{aligned}$$

we have

$$M_{n} - \sum f^{p}(\xi_{i}) W_{n}(\beta_{i}) \leq C_{7} \frac{2\pi}{K ^{p}} M_{n}, $$

from which it follows that

$$M_{n} - \sum f^{p}(\xi_{i}) W_{n}(\beta_{i}) \leq\frac{1}{2} M_{n}, $$

or, equivalently,

$$\frac{1}{2} M_{n} \leq\sum f^{p}( \xi_{i}) W_{n}(\beta_{i}), $$

provided

$$K \geq(C_{7} 4\pi)^{1/p} + \frac{\log_{2}L}{p}+6. $$

Using

$$L^{-1}W_{n}(\beta_{i}) \leq W_{n}( \eta_{i}) \leq L W_{n}(\beta_{i}), $$

uniformly whenever $\eta_{i} \in J_{i}$, we have, for any $\xi_{i}$, $\eta_{i} \in J_{i}$,

$$\frac{1}{2L} M_{n} \leq\sum f^{p}( \xi_{i}) W_{n}(\eta_{i}). $$

In particular, this is true for the points $\gamma_{i} \in J_{i}$ and $\delta_{i} \in J_{i}$ where $f(\gamma_{i})=\min_{x \in J_{i}}f(x)$ and $W_{n}(\delta_{i})=\min_{x \in J_{i}}W_{n}(x)$; hence, we have, for any $x_{i} , y_{i} \in J_{i} $,

$$\begin{aligned} \frac{1}{2L} M_{n} =&\frac{1}{2L}\sum f^{p}(\alpha_{i}) W_{n}(\beta _{i}) \\ \leq& \sum f^{p}(x_{i}) W_{n}(y_{i}) \\ \leq& M_{n} \leq2L\sum f^{p}( \gamma_{i}) W_{n}(\delta_{i}). \end{aligned}$$

If we also note that $y_{i} \in J_{i}$ implies

$$n \int_{J_{i}} W(x)\,dx \leq W_{n}(y_{i}) \leq L^{(\log_{2}K)} n \int_{J_{i}} W(x)\,dx, $$

it follows that

2.10 $$\begin{aligned}& \frac{n}{2L}\sum \int_{J_{i}}f^{p}(\alpha_{i})W(x)\,dx \\& \quad \leq \sum f^{p}(x_{i}) W_{n}(y_{i}) \\& \quad \leq 2L L^{(\log_{2}K)} n \sum \int_{J_{i}} f^{p}(\gamma _{i})W(x)\,dx, \end{aligned}$$

whenever $x_{i},y_{i} \in J_{i}$. Letting $x_{i}=y_{i}=2i\pi/(Kn ^{*}) +x$ and integrating the above inequality with respect to $x \in[0, (2\pi)/(Kn^{*})]$, we obtain

$$\begin{aligned} \frac{\pi}{L K}\sum \int_{J_{i}}f^{p}(\alpha_{i})W(x)\,dx \leq& \sum \int_{J_{i}} f^{p}(x) W_{n}(x)\,dx \\ \leq& \frac{8\pi L L^{(\log_{2}K)} }{K} \sum \int_{J_{i}}f^{p}( \gamma_{i})W(x)\,dx. \end{aligned}$$

Since $f(\alpha_{i})=\max_{x \in J_{i}}f(x)$ and $f(\gamma_{i})= \min_{x \in J_{i}}f(x)$, we obtain

$$\begin{aligned} \frac{\pi}{L K}\sum \int_{J_{i}}f^{p}(x)W(x)\,dx \leq& \sum \int_{J_{i}} f^{p}(x) W_{n}(x)\,dx \\ \leq& \frac{8\pi L L^{(\log_{2}K)}}{K} \sum \int_{J_{i}} f^{p}(x)W(x)\,dx, \end{aligned}$$

which proves the theorem. □

## Results on weighted inequalities for generalized trigonometric polynomials with doubling weights

In this section we apply the basic theorem to prove the weighted inequalities for generalized trigonometric polynomials with doubling weights.

### Bernstein inequality

Bernstein type inequalities have numerous applications in approximation theory. The following is a Bernstein type inequality for generalized trigonometric polynomials with doubling weights.

#### Theorem 3.1

*Let* *W* *be a doubling weight and let* $0< p<\infty$. *Then there is a constant* $C>0$ *depending only on* *p* *and on the weight* *W* *such that for every* $f \in\mathbb{GT}_{n}$ ($1 \leq n \in\mathbb{R}^{+}$) *with each* $r_{j} \geq2$ *in its representation* (1.1) *we have*

3.1 $$ \int_{-\pi}^{\pi} \bigl\vert f'\bigr\vert ^{p} W \leq C n^{p} \int_{- \pi}^{\pi} \vert f\vert ^{p} W. $$

#### Proof

By Theorem 2.1 we can replace $W_{n}$ by *W* in (2.6). □

### Marcinkiewicz inequality

A Marcinkiewicz type inequality is useful when we need to estimate $L_{p}$ norms of a trigonometric polynomials by a finite sum. The following theorem describes such inequalities for generalized trigonometric polynomials with doubling weights.

#### Theorem 3.2

*Let* *W* *be a doubling weight and let* $0< p<\infty$. *Then there are two constants* $K>0$ *and* $C>0$ *depending only on* *p* *and on the weight* *W* *such that for every* $f \in\mathbb{GT}_{n}$ ($1 \leq n \in \mathbb{R}^{+}$) *with each* $r_{j} \geq2$ *in its representation* (1.1) *we have*

$$\int_{-\pi}^{\pi} f^{p} W \leq \frac{C}{n}\sum_{j=0}^{m}f ^{p}(\tau_{j})W_{n}(\tau_{j}) $$

*provided the points* $\tau_{0}<\tau_{1}<\cdots<\tau_{m}$ *satisfy* $\tau_{j+1}-\tau_{j} \leq2\pi/(Kn)$ *and* $\tau_{m}\geq\tau_{0} + 2 \pi$.

#### Proof

Let $n^{*}=[n]$. In the proof of Theorem 2.1 we have proved in (2.10) that there exists a positive integer *K* such that if $J_{i}= [ \frac{2i\pi}{Kn^{*}}, \frac{2(i+1)\pi}{ Kn^{*}} ] $, $i=0, 1, \ldots, Kn^{*}-1$, and $x_{i} \in J_{i}$ arbitrary, then

$$\frac{n}{2L}\sum \int_{J_{i}}f^{p}(\alpha_{i})W(x)\,dx \leq \sum f^{p}(x _{i}) W_{n}(x_{i}). $$

Since $f(\alpha_{i})=\max_{x \in J_{i}}f(x)$, we have

$$\frac{n}{2L}\sum \int_{J_{i}}f^{p}(x)W(x)\,dx \leq\sum f^{p}(x_{i}) W _{n}(x_{i}). $$

Thus, the theorem is true if there is at least one point $\tau_{j}$ ($\operatorname{mod}\ 2\pi$) in every $J_{i}$, $i=0, 1, \ldots, K n^{*}-1$, or if the points $\tau_{0}<\tau_{1}<\cdots<\tau_{m}$ satisfy $\tau _{j+1}-\tau _{j} \leq2\pi/(Kn)$ and $\tau_{m}\geq\tau_{0} + 2\pi$. □

### Schur inequality

The following is a Schur type inequality for generalized trigonometric polynomials with doubling weights involving generalized Jacobi weights.

#### Theorem 3.3

*Let* *W* *be a doubling weight and let* $0< p<\infty$. *Let* *V* *be a generalized Jacobi weight of the form*

$$V(x)= v(x) \prod_{i=1}^{m}\vert x-x_{i}\vert ^{\gamma_{i}} ,\quad x_{i}, x \in[- \pi, \pi), \gamma_{i}>0 , $$

*where* *v* *is a positive measurable function bounded away from* 0 *and* ∞. *Then there is a constant* $C>0$ *independent of* *n* *such that for every* $f \in\mathbb{GT}_{n}$ ($1\leq n \in\mathbb{R}^{+}$) *with each* $r_{j} \geq2$ *in its representation* (1.1) *we have*

$$\int_{-\pi}^{\pi} f^{p} W \leq C n^{\Gamma} \int_{-\pi}^{\pi} f^{p} W V , $$

*where* $\Gamma=\max_{1\le i \le m}\{\gamma_{i}\} $.

#### Proof

By the Lemma 4.5 in [2], *WV* is also a doubling weight and it is easy to see that $(WV)_{n}(x) \sim W_{n}(x)V_{n}(x)$ and $V_{n}(x) \geq cn^{-\Gamma}$. Thus, by Theorem 2.1, we have

$$\begin{aligned} \int_{-\pi}^{\pi} f^{p} W V \sim& \int_{-\pi}^{\pi} f ^{p} (W V)_{n} \\ \sim& \int_{-\pi}^{\pi} f^{p} W_{n} V_{n} \\ \geq& \frac{c}{n^{\Gamma}} \int_{-\pi}^{\pi} f^{p} W_{n} \\ \sim& \frac{1}{n^{\Gamma}} \int_{-\pi}^{\pi} f^{p} W, \end{aligned}$$

which completes the proof. □

## Results on weighted inequalities for generalized trigonometric polynomials with $A_{\infty}$ weights

In this section we prove the weighted inequalities for generalized trigonometric polynomials with $A_{\infty}$ weights.

### Remez inequality

The Remez inequality is useful because we can exclude exceptional sets of measure at most 1. The following describes such inequalities for generalized trigonometric polynomials with $A_{\infty}$ weights.

#### Theorem 4.1

*Let* $0< p<\infty$ *and let* *W* *be an* $A_{\infty}$ *weight*. *Then there is a constant* $C>0$ *depending only on* *p* *and on the weight* *W* *such that if* $f \in\mathbb{GT}_{n}$ ($1 \leq n \in\mathbb{R}^{+}$) *with each* $r_{j} \geq2$ *in its representation* (1.1) *and* *E* *is a measurable subset of* $[0, 2\pi]$ *of measure at most* $\lambda\in(0, 1]$, *then*

4.1 $$ \int_{[0, 2\pi]} f^{p} W \leq C^{1+n\lambda} \int_{[0, 2\pi]\setminus E} f^{p} W. $$

#### Proof

First we show that if we replace *W* by $W_{n}$ in (4.1), then inequality holds. By (2.3), we have a trigonometric polynomial $P_{n}$ of degree at most $( \frac{\log_{2} L}{p} +4) n$ such that

$$P_{n}^{p} \sim W_{n} . $$

Then we apply the Remez inequality for generalized trigonometric polynomials (see Theorem 8 in [1]) to $fP_{n} \in\mathbb{GT} _{bn}$ where $b= ( \frac{\log_{2} L}{p} +5) $ as follows:

4.2 $$\begin{aligned} \int_{[0, 2\pi]} f^{p} W_{n} \leq& C_{1} \int_{[0, 2\pi]} (f P _{n})^{p} \\ \leq& C_{1} C^{1+bn\lambda} \int_{[0, 2\pi]\setminus E} (f P_{n})^{p} \\ \leq& C_{1}^{2} C^{1+bn\lambda} \int_{[0, 2\pi]\setminus E} f^{p} W_{n} . \end{aligned}$$

Note that the case $1 < p < \infty$ of the theorem follows from the case $0< p\leq1$. So from now on we assume that $0< p\leq1$. Next we follow the proof of Theorem 2.1. Let *K* be a large positive even integer which will be chosen later, and set $n^{*} = [n]$ and

$$J_{i} := \biggl[ \frac{2i\pi}{Kn^{*}}, \frac{2(i+1)\pi }{Kn^{*}} \biggr] ,\quad i=0, 1, \ldots, Kn^{*} -1. $$

Define the set *J* by

$$J:=\bigl\{ i : \vert E\cap J_{i}\vert \geq \vert J_{i}\vert /2 \bigr\} , $$

and let

$$I^{*} = \bigcup_{i \in J} J_{i} . $$

Then

$$\bigl\vert I^{*}\bigr\vert \leq2\sum _{i \in J}\vert E \cap J_{i} \vert \leq2\vert E \vert \leq2\lambda. $$

Let $\alpha_{i} \in J_{i}$ be a point such that $f(\alpha_{i}) = \max_{x \in J_{i}} f(x)$ and let $\beta_{i} \in J_{i}$ be a point such that $W_{n}(\beta_{i})=\max_{x \in J_{i}} W_{n}(x)$. Let

$$M_{n}= \sum_{i \notin J} f^{p}( \alpha_{i}) W_{n}(\beta_{i}). $$

Now let $\xi_{i} \in J_{i}$ be arbitrary. Using exactly the same method as in the proof of Theorem 2.1 (from (2.7) to (2.9)), we have

$$M_{n} - \sum_{i \notin J} f^{p}( \xi_{i}) W_{n}(\beta_{i}) \leq C_{2} K^{1-p} n \int_{0}^{2\pi} f^{p} W_{n}. $$

By (4.2) we have

$$\begin{aligned} \int_{0}^{2\pi} f^{p} W_{n} \leq& C_{1}^{2} C^{1+bn 2\lambda} \int_{[0, 2\pi]\setminus I^{*}} f^{p} W_{n} =C_{1}^{2} C^{1+bn 2 \lambda} \sum _{i \notin J} \int_{J_{i}} f^{p} W_{n} \\ \leq& C_{1}^{2} C^{1+bn 2\lambda} \sum _{i \notin J}\vert J_{i}\vert f^{p}( \alpha_{i})W_{n}(\beta_{i}) =C_{1}^{2} C^{1+bn 2\lambda}\frac{2 \pi}{Kn^{*}}M_{n}, \end{aligned}$$

hence,

$$M_{n} - \sum_{i \notin J} f^{p}( \xi_{i}) W_{n}(\beta_{i}) \leq C_{3} C ^{1+bn 2\lambda} \frac{1}{K^{p}} M_{n}, $$

from which it follows that

$$M_{n} - \sum_{i \notin J} f^{p}( \xi_{i}) W_{n}(\beta_{i}) \leq \frac{1}{2} M_{n}, $$

provided

4.3 $$ K = \bigl(2C_{3} C^{1+2bn\lambda}\bigr)^{1/p}. $$

Using

$$L^{-1}W_{n}(\beta_{i}) \leq W_{n}( \eta_{i}) \leq L W_{n}(\beta_{i}) $$

uniformly whenever $\eta_{i} \in J_{i}$, we have, for any $\xi_{i}$, $\eta_{i}\in J_{i}$,

$$\frac{1}{2L} M_{n} \leq\sum_{i \notin J} f^{p}(\xi_{i}) W_{n}(\eta _{i}). $$

In particular, this is true for the points $\gamma_{i} \in J_{i}$ and $\delta_{i} \in J_{i}$ where $f(\gamma_{i})=\min_{x \in J_{i}}f(x)$ and $W_{n}(\delta_{i})=\min_{x \in J_{i}}W_{n}(x)$, hence, we have, for any $x_{i} , y_{i} \in J_{i} $,

$$\begin{aligned} \frac{1}{2L} M_{n} \leq& \sum_{i \notin J} f^{p}(x_{i}) W_{n}(y_{i}) \\ \leq& M_{n} \leq2L\sum_{i \notin J} f^{p}(\gamma_{i}) W_{n}(\delta _{i}). \end{aligned}$$

Now we use the property of the $A_{\infty}$ weight. If $i \notin J$, then $\vert J_{i} \setminus E\vert \geq \vert J_{i}\vert /2= \pi/(Kn^{*})$, hence, by Lemma 5.1(vi)′ in [2], there are constants *s* and *D* such that, for $y_{i} \in J_{i}$, $i \notin J$,

4.4 $$\begin{aligned} W_{n}(y_{i})=n \int_{y_{i} -1/n}^{y_{i} +1/n} W \leq& n D \biggl( \frac{2/n}{\vert J_{i} \setminus E\vert } \biggr) ^{s} \int_{J_{i} \setminus E} W \\ \leq& n D \biggl( \frac{2K}{\pi} \biggr) ^{s} \int_{J_{i} \setminus E} W. \end{aligned}$$

Similarly to (2.10), we have

$$\sum_{i \notin J} f^{p}(x_{i}) W_{n}(y_{i}) \leq C_{4} n D \biggl( \frac{2K}{ \pi} \biggr) ^{s} \sum_{i \notin J} \int_{J_{i} \setminus E} \Bigl( \min_{x \in J_{i}} f^{p}(x) \Bigr) W(x)\,dx, $$

whenever $x_{i}$, $y_{i} \in J_{i}$. Letting $x_{i}=y_{i}=2i\pi/(Kn ^{*}) +x$ and integrating the above inequality with respect to $x \in[0, (2\pi)/(Kn^{*})]$, we obtain

$$\begin{aligned} \sum_{i \notin J} \int_{J_{i}} f^{p}(x)W_{n}(x)\,dx =& \int_{[0, 2\pi] \setminus I^{*}}f^{p} W_{n} \\ \leq& \biggl( \frac{2\pi}{Kn^{*}} \biggr) C_{4} n D \biggl( \frac{2K}{ \pi} \biggr) ^{s} \sum_{i \notin J} \int_{J_{i} \setminus E} \Bigl( \min_{x \in J_{i}} f^{p}(x) \Bigr) W(x)\,dx \\ \leq& C_{4} 2^{1+s} D \biggl( \frac{\pi}{K} \biggr) ^{1-s} \sum_{i \notin J} \int_{J_{i} \setminus E} f^{p}(x) W(x)\,dx \\ \leq& C_{5} K^{s-1} \int_{[0, 2\pi] \setminus(I^{*} \cup E)} f ^{p}(x) W(x)\,dx. \end{aligned}$$

Applying Theorem 2.1, (4.2), and the definition of *K* in (4.3), we have

$$\begin{aligned} \int_{[0, 2\pi]}f^{p} W \leq& C_{6} \int_{[0, 2\pi]}f^{p} W_{n} \leq C_{6} C_{1}^{2} C^{1+2bn\lambda} \int_{[0, 2\pi]\setminus I^{*}}f ^{p} W_{n} \\ \leq& C_{7} C^{1+2bn\lambda}K^{s-1} \int_{[0, 2\pi]\setminus(I^{*}\cup E) }f^{p} W \\ \leq& C_{8} \bigl( C^{(p+s-1)/p} \bigr) ^{1+2bn\lambda} \int_{[0, 2\pi]\setminus E }f^{p} W, \end{aligned}$$

which proves the theorem. □

### Nikolskii inequality

Nikolskii inequality is used to compare the $L_{p}$ and $L_{q}$ norms of polynomials. The following theorem describes such inequalities for generalized trigonometric polynomials with $A_{\infty}$ weights.

#### Theorem 4.2

*Let* *W* *be an* $A_{\infty}$ *weight and let* $0< p< q<\infty$. *Then there is a constant* $C>0$ *depending only on* *p* *and* *q* *and on the weight* *W* *such that for every* $f \in\mathbb{GT}_{n}$ ($1 \leq n \in \mathbb{R}^{+}$) *with each* $r_{j} \geq2$ *in its representation* (1.1) *we have*

$$\biggl( \int_{-\pi}^{\pi} f^{q} W \biggr) ^{1/q} \leq C n^{1/p -1/q} \biggl( \int_{-\pi}^{\pi} f^{p} W^{p/q} \biggr) ^{1/p}. $$

#### Proof

Define the set *E* by

$$E= \biggl\{ x \in[-\pi, \pi] : f^{q}(x)W(x) \geq n \int_{- \pi}^{\pi}f^{q} W \biggr\} . $$

Then $\int_{E} f^{q} W \geq n \vert E\vert \int_{-\pi}^{\pi}f^{q} W$, hence, $\vert E\vert \leq1/n$. Now applying the Theorem 4.1, we have

$$\begin{aligned} \int_{-\pi}^{\pi}f^{q} W \leq& C \int_{[-\pi, \pi] \setminus E}f ^{q} W \\ \leq& C \bigl\Vert f^{q} W\bigr\Vert _{L_{[-\pi, \pi] \setminus E }^{\infty}}^{(q-p)/q} \biggl( \int_{-\pi}^{\pi}f^{p} W^{p/q} \biggr) \\ \leq& C n^{(q-p)/q} \biggl( \int_{-\pi}^{\pi}f^{q} W \biggr) ^{(q-p)/q} \biggl( \int_{-\pi}^{\pi}f^{p} W^{p/q} \biggr). \end{aligned}$$

Taking *p*th root yields the theorem. □

## Conclusions

In this paper, we have established weighted inequalities, such as the Bernstein, Marcinkiewicz, Schur, Remez, Nikolskii inequalities, for generalized trigonometric polynomials with doubling weights. We also have established the large sieve for generalized trigonometric polynomials with doubling weights.

## Footnotes

1 Here, and in what follows, $[x]$ denotes the integer part of *x*.

2 In what follows $A \sim B$ means that there are two positive constants $C_{1}$ and $C_{2}$ such that $C_{1} \leq B/A \leq C_{2}$.
